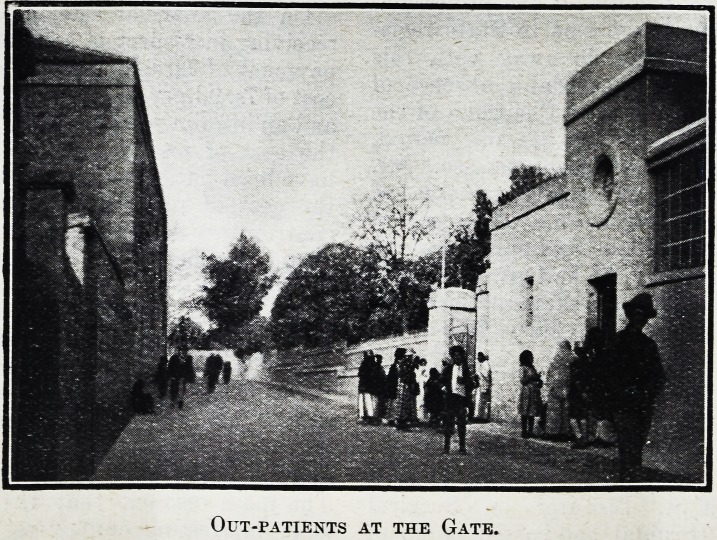# Centenary of Medical Missions

**Published:** 1924-01

**Authors:** 


The Jerusalem Mission Hospital.

### CENTENARY OF MEDICAL MISSIONS.

### WORK OF THE JERUSALEM HOSPITAL.

THE opening of the Jerusalem Post of the London Mission to the Jews in the closing month of 1823 really marked the beginnings of modern medical missions. This centenary was remembered in many churches on Sunday, December 9, which was also the anniversary of General Allenby's deliverance of Jerusalem in the Great War.

### Joseph Wolff as Pioneer.

In consequence of the hopeful accounts brought by Joseph Wolff, a man of intense missionary fervour, himself a Jew, and the father of the late Sir Henry Drummond Wolff, the Society sent out the Rev. Lewis Way, accompanied by the Rev. W. B. Lewis, to Palestine in 1823. The former fell ill and had to return, but Mr. Lewis entered Jerusalem on December 13, 1823, and established a Post there. Medical work almost immediately became part of the activities of the mission, and Dr. Dalton, an Irish practitioner, went out to Jerusalem, where, shortly afterwards, he died.

### A Great Eastern Hospital.

The work was continued in face of many difficulties by Dr. MacGowan and Dr. Chaplin, who accomplished a splendid work and put the Medical Mission on a firm foundation. It was in 1885 that Dr. Percy d'Erf Wheeler, a St. Thomas's Hospital man and a member of an Irish family, who had intended entering the Army, offered to take over the work of the hospital. The demands for the services of the hospital from Jews were so great that extension soon became inevitable. Dr. Wheeler hoped to get £5,000 from his Committee, but they were unable at that time to grant this sum. He therefore set about raising it himself, and was so successful that almost entirely through his own efforts and those of his friends, notably Lady Meath, the family of Richard Cadbury and many at Norwich, there is standing, to-day, one of the finest hospitals in the Near East, built at a cost of £20,000.

### Catching the Bird on the Wing.

It was with characteristic impetuosity and energy that at forty-eight hours' notice Dr. Wheeler took advantage of the late Earl of Northbrook's presence in Jerusalem in 1895 to get him to lay the foundation stone of the building designed by Professor Beresford Pite. This is on the detached pavilion plan and consists of a central administration block, four wards, dispensary and out-patients' department. Subsequent additions were made through help from Ireland. The nursing staff, consisting chiefly of English trained sisters, has represented almost as many nationalities and religious faiths as the patients; among the nurses, when Dr. Wheeler left, were an English matron, Miss Wells, several Jewish girls, a Sudanese Christian, an Armenian, and a Moslem girl. Dr. Wheeler is proud of the fact that the Turkish authorities were so struck with the hospital that they have begun to build a replica in the Holy City of Medina. Dr. Wheeler is now in England after a breakdown in health, and the hospital is in charge of Dr. Orr Ewing, with Dr. K. W. Kingdon as assistant and Miss E. M. Barber (late of St. Bartholomew's Hospital) as Matron. The number of patients dealt with by the hospital averages 1,500 in-patients and 6,000 out-patients, and there is a staff of fifteen.

Out-patients at the Gate.

## Figures and Tables

**Figure f1:**
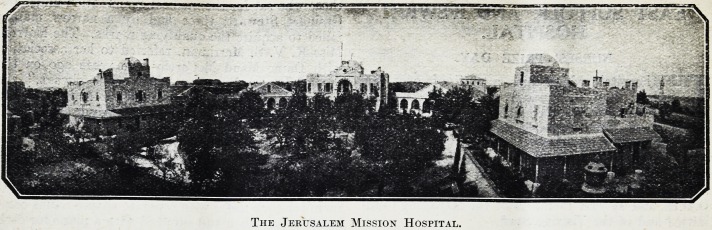


**Figure f2:**